# *Kalanchoe crenata* Haw. (Crassulacea) Decreases Hippocampal Neuron Loss and Improves Memory and Executive Function in Aged Rats: Implications for Anti‐Inflammatory and Antioxidant Mechanisms

**DOI:** 10.1002/brb3.70261

**Published:** 2025-02-16

**Authors:** Antoine Kavaye Kandeda, Christophe Mezui, Sandry Kengni, Ndeva Baldagai, Symphorien Talom Mabou

**Affiliations:** ^1^ Department of Animal Biology and Physiology, Faculty of Science University of Yaoundé I Yaoundé Cameroon; ^2^ Department of Biotechnology and Pharmacognosy Faculty of Science, University of Ebolowa Ebolowa Cameroon; ^3^ Department of Microbiology, Parasitology, Hematology, and infectious diseases Faculty of Medicine and Biomedical Sciences, University of Yaoundé I Yaoundé Cameroon

**Keywords:** antiamnesic, anti‐inflammatory, antioxidant, *Kalanchoe crenata*

## Abstract

**Introduction:**

Alzheimer's disease is a neurodegenerative disease that alters learning and memory processes. *Kalanchoe crenata* (Crassulaceae) has long been used in Cameroonian traditional medicine to treat hypertension, malaria, and dementia. The present study aims to evaluate the anti‐amnesic effect of an aqueous extract of *K. crenata* in D‐galactose‐treated rats and possible mechanisms of action.

**Methods:**

Memory impairment was induced in rats by subcutaneous injection of D‐galactose (350 mg/kg) once daily for 30 days. At the end of the procedure, the animals were assessed for memory impairment using Morris water maze and object recognition tasks. Animals with memory impairment were divided into six groups of eight rats each and treated once daily for 24 days as follows: the negative control group received per os distilled water (10 mL/kg); the positive control group received donepezil (2 mg/kg, p.o.); and three test groups received the extract of *K. crenata* (62, 124, and 248 mg/kg, p.o.). A group of eight rats was added and served as a control group. After completion of the procedure, the memory deficit in rats was reassessed by the object recognition test on Day 15 of the treatment, the Morris water maze test on Day 18, and the open‐field test on Day 24. At the end of behavioral experiments, the animals were sacrificed and some biochemical parameters in the hippocampus were estimated. In addition, histological analysis of the hippocampus was performed.

**Results:**

*K. crenata* significantly decreased the time to reach the platform and increased the time spent in the target quadrant of the Morris water maze. It also increased the discrimination index during the object recognition test. The extract significantly reversed D‐galactose‐induced oxidative stress and inflammation. This was confirmed by the attenuation of neuronal loss.

**Conclusion:**

These findings suggest that *K. crenata* extract possesses an anti‐amnesic‐like effect probably mediated by antioxidant and anti‐inflammatory mechanisms.

## Graphical abstract

Aqueous extract of *Kalanchoe crenata* has the potential to cure Alzheimer's disease.

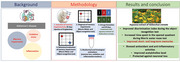

## 1 Introduction

Alzheimer's disease (AD) is an irreversible and progressive brain disorder. It is the most common form of dementia (Zvěřová 2019). The global prevalence of AD is approximately 46.8 million people with 7.7 million new cases per year (Prince et al. 2015). This number could increase every 20 years and reach 74.7 million by 2030 (Prince et al. 2016; 2015). The prevalence of AD in Cameroon is unknown as approximately 90% of people with dementia go undiagnosed (Akinyemi et al. 2022; Lekoubou, Echouffo‐Tcheugui, and Kengne 2014). The mechanisms underlying the pathophysiology of AD are complex and remain unclear (Kumar, Singh, and Ekavali 2015; Mufson et al. 2016). However, many reports suggest that AD is characterized by extracellular deposits of amyloid β‐protein (Aβ), the occurrence of neurofibrillary tangles, cholinergic neurotransmission deficiency, and neuronal loss (Kar et al. 2004). Aβ deposits in the hippocampus cause neuronal death through multiple mechanisms including oxidative stress, inflammation, and apoptosis (Cai, Zhao, and Ratka 2011; Chen et al. 2020). Clinically, AD is characterized by cognitive impairment associated with aphasia, apraxia, and agnosia (Monteiro et al. 2023). Currently, no drug treatment can cure AD (Briggs, Kennelly, and O'Neill 2016). In addition, these drugs are associated with numerous adverse effects (Samanta et al. 2006). Therefore, medicinal plants are an alternative to discovering effective and safe treatments for AD. *Kalanchoe crenata* (Crassulaceae) grows in tropical and Subtropical Africa (Smith 2022). In Africa, traditional healers use *K. crenata* extract to treat asthma, otitis, male infertility, gastric ulcers, dementia, and neuropathic pain (Nguelefack et al. 2006). Phytochemical analysis of the aqueous leaf extract revealed the presence of polysaccharides, anthraquinones, terpenes, alkaloids, flavonoids, polyphenols, saponins, and tannins (Milad, El‐Ahmady, and Singab 2014). These metabolites have been shown to possess analgesic, anticonvulsant, anti‐inflammatory, antioxidant, and anti‐diabetic properties (Foyet 2012; Kablan, Adiko, and Abrogoua 2008); Kamgang et al. 2008; Théophile et al. 2006). To our knowledge, anti‐amnesic like‐effect of the extract of *K. crenata* leaves has not been studied. The aim of this research was therefore to evaluate anti‐amnesic like‐effect of *K. crenata* extract on D‐galactose treated rats. The involvement of anti‐inflammatory and antioxidant mechanisms was also investigated.

## 2 Materials and Methods

### 2.1 Plant Collection and Identification

**FIGURE 1:**
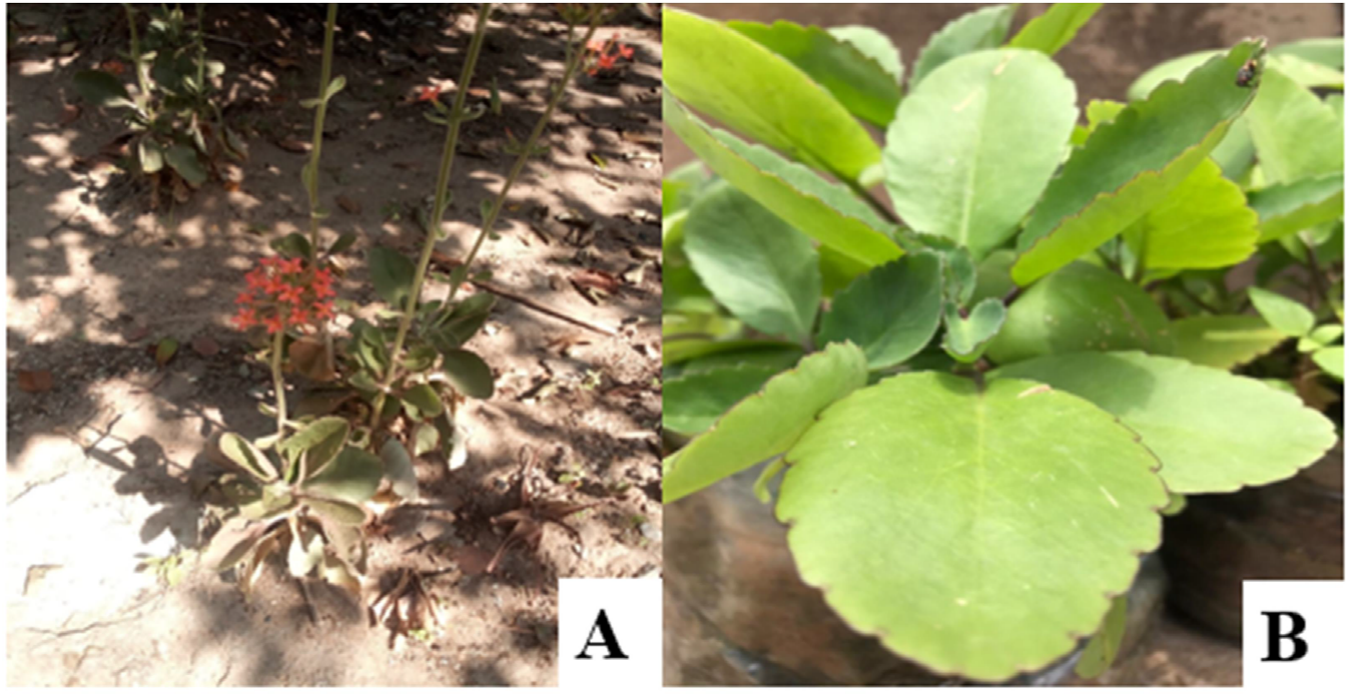
*Kalanchoe crenata* whole plant (A) and leaves (B).

Fresh leaves of the plant were collected in West Cameroon, and identified as *K. crenata* Haw. (Crassulacea) in the Cameroon National Herbarium under number 35196∕HNC (Figure 1). The plant name was checked at https://powo.science.kew.org/taxon/urn:lsid:ipni.org:names:274311‐1. The extract of *K. crenata* was prepared by imitating the method of traditional healers. Briefly, fresh *K. crenata* leaves were harvested, washed, shade‐dried for 30 days, and grounded to a powder. A total of 400 grams of powder were boiled in 3 L of distilled water. After cooling to room temperature (24°C –27°C), the mixture was filtered through Whatman No. 3 paper. The filtrate was freeze‐dried and dry matter (39.09 g) was obtained, giving a yield of 9.77%. Doses of 62, 124, and 248 mg/kg were used in this study.

### 2.2 Animals and Ethics

Male Wistar rats aged 13–14 weeks were used. These animals were raised in the animal facility of the Laboratory of Animal Physiology (University of Yaoundé I‐Cameroon) under natural conditions of temperature (24°C–27°C) and light. The animals were acclimatized to laboratory conditions before their use in experiments. All procedures were performed according to the guidelines of the National Ethics Committee of Cameroon (Ref. No. FW‐IRB00001954, October 22, 1987).

### 2.3 Drugs and Chemicals

Ketamine, diazepam, donepezil, and D‐galactose were obtained from Sigma Chemical Co, St. Louis (USA), while Tris‐HCl buffer (pH 7.4) was obtained from Rhone‐Poulenc and Sigma Tech laboratories (France), Trichloroacetic acid (TCA), thiobarbituric acid (TBA), and formaldehyde from Prost Pharma (France). Furthermore, proinflammatory cytokine assay kits were obtained from Quantikine (Biotechne Inc., Minneapolis, USA).

### 2.4 Experimental Design

Behavioral experiments in the present study were performed in two sets:

#### 2.4.1 Induction Phase

**FIGURE 2:**
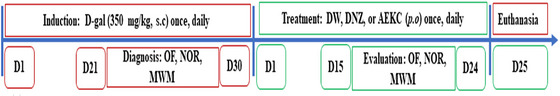
Experimental design. D‐gal, d‐galactose; DW, distilled water (10 mL/kg); DNZ, Donepezil (2 mg/kg, p.o.); AEKC, aqueous extract of *K. crenata* (62, 124, and 248 mg/kg); OF, open‐field test; NOR, Novel object recognition test; MWM, Morris water maze test; D, day; p.o., per os; s.c., subcutaneous.

Memory impairment was induced in 50 rats by subcutaneous injection (s.c.) of D‐galactose (D‐gal) (350 mg/kg), once daily for 30 days. A group of eight normal rats was, however, treated with distilled water (10 mL/kg, s.c.), once a day for 30 days (Figure 2). This group served as a control group. On Day 21 of the induction, memory deficit was assessed by the object recognition test; this task took place over 3 days (Day 21 to Day 23). After 24 h, that is, on Day 24 of the induction, the Morris maze task (MM) was performed in 6 days (Day 24 to Day 29). Finally, on Day 30, that is, 24 h after the MM, the open‐field test was performed (Figure 2).

#### 2.4.2 Treatment Phase

After completing behavioral tests, animals (*n* = 42) with memory deficits were selected for further testing. These animals were randomly divided into five groups of eight animals each and treated once daily for 24 days as follows:

– the negative control group (D‐gal + DW group) received per os (p.o.) distilled water (10 mL/kg);
– the positive control group (D‐gal + DNZ group) received donepezil (2 mg/kg, p.o.);
– three test groups (D‐gal + KC groups) received an aqueous extract of *K. crenata* (62, 124, and 248 mg/kg).

The control group (DW + DW group) above was treated with distilled water (10 mL/kg, p.o.).

On the Day 15 of treatment, the rats underwent an object recognition test. The test was completed in 3 days. After 24 h, that is, on Day 18, the animals were subjected to the MM, which lasted 6 days. After 24 h of the MM, that is, on Day 24, the open‐field test was performed in one day (Figure 2). After behavioral tests, the animals were euthanized, and their hippocampi and blood were collected for further biochemical and histological analysis.

### 2.5 Behavioral Observations and Tests

#### 2.5.1 Object Recognition Task

The object recognition task (ORT) relies on the natural propensity of rodents to explore a novel object rather than a familiar one. It allows the assessment of recognition memory in 3 days. This test was done in three phases: habituation, acquisition, and retention (Antunes and Biala 2012; Kandeda, Nguedia et al. 2021). On Day 1 of the experiment (habituation phase, i.e., and phase 1), animals were permitted to explore the maze (without any object) for 5 min to avoid neophobia stress (Antunes and Biala 2012; Kandeda, Nguedia et al. 2021). The second phase started 24 h later. Two identical (X + X) objects, opposite to each other, were placed in the corners of the paradigm (Lissner et al. 2021). Consequently, the time taken by each rat to explore the objects was recorded (Antunes and Biala 2012). The retention phase occurred 24 h after the acquisition phase and lasted 5 min (Kouémou et al. 2017). This phase is analogous to the acquisition phase, with the exception that one of the objects (X) has been substituted by a novel object (Y) (Cole et al. 2019). The time to explore the familiar object (tX) and the time to explore the novel object (tY) were recorded (Antunes and Biala 2012). The discrimination index (DI) was calculated as follows: [tY − tX/tY + tX] (Antunes and Biala 2012).

#### 2.5.2 Morris Water Maze Task

The MM is used to study the impairment of spatial memory. This test is associated with the innate tendency of animals to escape from water (Othman, Hassan, and Che Has 2022). A tank (61 cm high and 151 cm in diameter) was filled with water (25°C) to 30 cm. The labyrinth was virtually partitioned into four quadrants of equal size in virtual terms: North, South, East, and West (Djeuzong et al. 2021). In the center of one quadrant, a white refuge pedestal (8 cm diameter by 29 cm high) was placed 1 cm below the water surface (Djeuzong et al. 2021). To prevent the platform from being visible on the water surface, the water was bleached by the addition of liquid milk (Djeuzong et al. 2021). The maze was in a room with various visual clues. The first day began with the habituation phase and each rat was acclimatized for 60 s without the platform. On Day 2, the acquisition phase took place over 4 days (Day 2–6) with three trials per day. Each trial did not last more than 150 s, meaning that each animal had 120 s to locate the platform. If the rat found the platform, it was allowed to remain on the platform for 10 s, but if the animal could not find the platform after 120 s, it was directed to the platform and permitted to remain there for 10 s. The time between trials was 5 min. The latency to find the platform was recorded for each animal (Vorhees and Williams 2006). Finally, the effectiveness of learning during the retention phase was evaluated (Vorhees and Williams 2006). During this phase, which lasted for 120 s, the platform was thus removed from the tank (Vorhees and Williams 2006). The time spent in the target quadrant and the latency to find the platform were thus recorded.

#### 2.5.3 Open‐Field Test

The open‐field test evaluates locomotor activity, exploration, and emotional reactivity in rodents (Bronikowski et al. 2001; Seibenhener and Wooten 2015). The maze used was made of wood (83 cm length × 83 cm width × 83 cm height). The exploration area was divided into 17 squares (21 cm × 21 cm) and one central square. The protocol used by Belzung (1999) is described here. Briefly, the animals were placed one after the other in the center of the paradigm. Each animal was therefore observed for 5 min. The following parameters were assessed: the number of crossings, the number of groomings, the number of rearings, the time in the central square, and the number of stools.

### 2.6 Biochemical Tests

#### 2.6.1 Euthanasia and Preparation of Homogenates

Immediately after testing, the animals were sacrificed under anesthesia with ketamine (70 mg/kg, i.p.)/diazepam (10 mg/kg, i.p.), and their blood was collected in dry tubes. The brain of each animal was removed and divided into two hemispheres. Hippocampi from the first half were isolated, washed with NaCl 0.9%, and blotted. They were weighed with a digital microbalance (U.S. Solid, USA) and homogenized with Tris‐HCl buffer (pH 7.4, 50 mM). The obtained supernatant was stored at −20°C following centrifugation (3000 rpm) at 4°C for 30 min for biochemical analyses (cholinergic, oxidative status, and neuro‐inflammation biomarkers). The second half was fixed in formalin 4% for histological analysis.

#### 2.6.2 Acetylcholinesterase and Butyrylcholinesterase Activity

The activity of acetylcholinesterase (AchE) and butyrylcholinesterase (BchE) was evaluated following the methodology described by Ellman (1959). In the test vial, 0.05 mL of supernatant, 3 mL of sodium phosphate buffer (pH 8), 0.1 mL of acetylthiocholine iodide, and 0.1 mL of 5, 5’dithio‐bis (2‐nitrobenzoic acid) were added. The absorbance was determined at 412 nm for 2 min at 30‐s intervals. Results were expressed as µM/min/mg of protein in tissue (1 µM/min/mg of AchE and BchE was defined as the amount of enzyme that hydrolyzed 1 µmol of acetylthiocholine and butyrylthiocholine iodide).

#### 2.6.3 Superoxide Dismutase Activity

The superoxide dismutase (SOD) activity was determined following the methodology described by Misra and Fridovich (Gaeta et al. 2002). To test vials, 134 µL of supernatant was added, while 1669 µL of phosphate buffer (0.06 M, pH 10.3) in the blank vials. Then, the reaction began when 200 µL of fresh adrenaline (0.29 mM) was added to all vials. After mixing was complete, the absorbance was read at 20 s and 80 s at 480 nm. SOD activity was determined in U/min/mg of tissue. One unit (U) of SOD was estimated as the quantity of SOD that inhibits 50% of adrenaline for 60 s.

#### 2.6.4 Reduced Glutathione Concentration

The concentration of reduced glutathione (GH) was determined according to Ellman's method (Ellman et al. 1961). Briefly, 1500 µL of 5′5‐dithiobis‐2‐nitrobenzoic acid (DNTB) and 600 µL of Tris‐HCl buffer (50 mM, pH 7.4) were added to blank vials containing 100 µL of Tris‐HCl buffer (50 mM, pH 7.4) and to test vials containing tissue homogenates (100 µL). The mixing solution was incubated for 1 h, and the absorbance was read at 412 nm against the blank. GH concentration was expressed as mmol/mg protein in tissue.

#### 2.6.5 Malondialdehyde Concentration

The method of Wilbur, Bernheim, and Shapiro (1949) was used to determine malondialdehyde (MDA) concentration. Briefly, distilled water (250 µL) and homogenate (20 µL) were added to the control vials and test vials, respectively. Then 250 µL of Tris‐HCl buffer (50 mM, pH 7.4), 500 µL of trichloroacetic acid (TCA, 20%), and 1000 µL of thiobarbituric acid (TBA, 0.67%) were added to each vial. The mixed solution was incubated in a water bath (90°C, 10 min), and after cooling at room temperature, it was centrifuged (3000 rpm, 15 min). The absorbance of the pink supernatant was measured against a blank at 530 nm. The concentration of MDA was defined in ρmol/g of tissue.

#### 2.6.6 Nitrite Concentration

The nitrogen monoxide (NO) content was determined by Griess's method (Grand, Guitton, and Goudable 2001). NO is a short half‐life compound that rapidly converts to nitrate (NO_3_^−^) and nitrite (NO_2_^−^). In this assay, the conversion of nitrate to nitrite is accompanied by color development in the presence of Griess's reagent. To estimate the amount of NO, 200 µL of homogenate and 200 µL of Griess reagent were added to test vials. The solution was mixed and the absorbance was read at 570 nm after 10 min. A standard NaNO_2_ curve was generated by a set of nitrite dilutions. The results were expressed in mmol/mL.

### 2.7 Proinflammatory Marker Assays

The proinflammatory cytokine assay (IL‐1β, IL‐6, TNF‐α, and IFN‐γ) was performed using the Enzyme‐Linked Immunosorbent Assay (ELISA) kit (Quantikine, Bio‐Techne, Minneapolis, USA).

### 2.8 Histopathological Analysis of Brain Tissue

Histological analysis was performed according to Survana, Layton, and Brancroft (2019). The slides were examined at 250X (CA1 and CA3 layers) and 100X (dentate gyrus) magnification with Scientico STM‐50 optical microscope (HSIDC Industrial Estate, Haryana, India). The microscope was equipped with a digital camera (Celestron 44421) connected to a computer. Furthermore, the density of CA1 and CA3 neurons was determined by counting the number of alive neurons per µm^2^; the Image J software (version 1.23) was thus used.

#### 2.8.1 Quantitative Phytochemical Assays

The levels of phenolic compounds, flavonoids, condensed tannins, total alkaloids, and total saponins were, respectively, quantified by methods of Hatami et al. (2014), Dehpour et al. (2009), Mang'Dobara et al. (2020), Ba et al. (2010), and Gracelin, Britto, and Kumar (2013).

### 2.9 Statistical Analysis

Analyses were performed with GraphPad Prism 8.0.1 for Windows (GraphPad Software, San Diego, California, USA) and Microsoft Office Excel 2019. Data were expressed as mean ± standard error of the mean. One‐way analysis of variance (ANOVA) compares the groups. When a difference exists, the Tukey post hoc test was used to separate them. Differences with *p* < 0.05 were considered significant.

## 3 Results

### 3.1 Effect of an Aqueous Extract of *K. crenata* on Object Recognition

**FIGURE 3:**
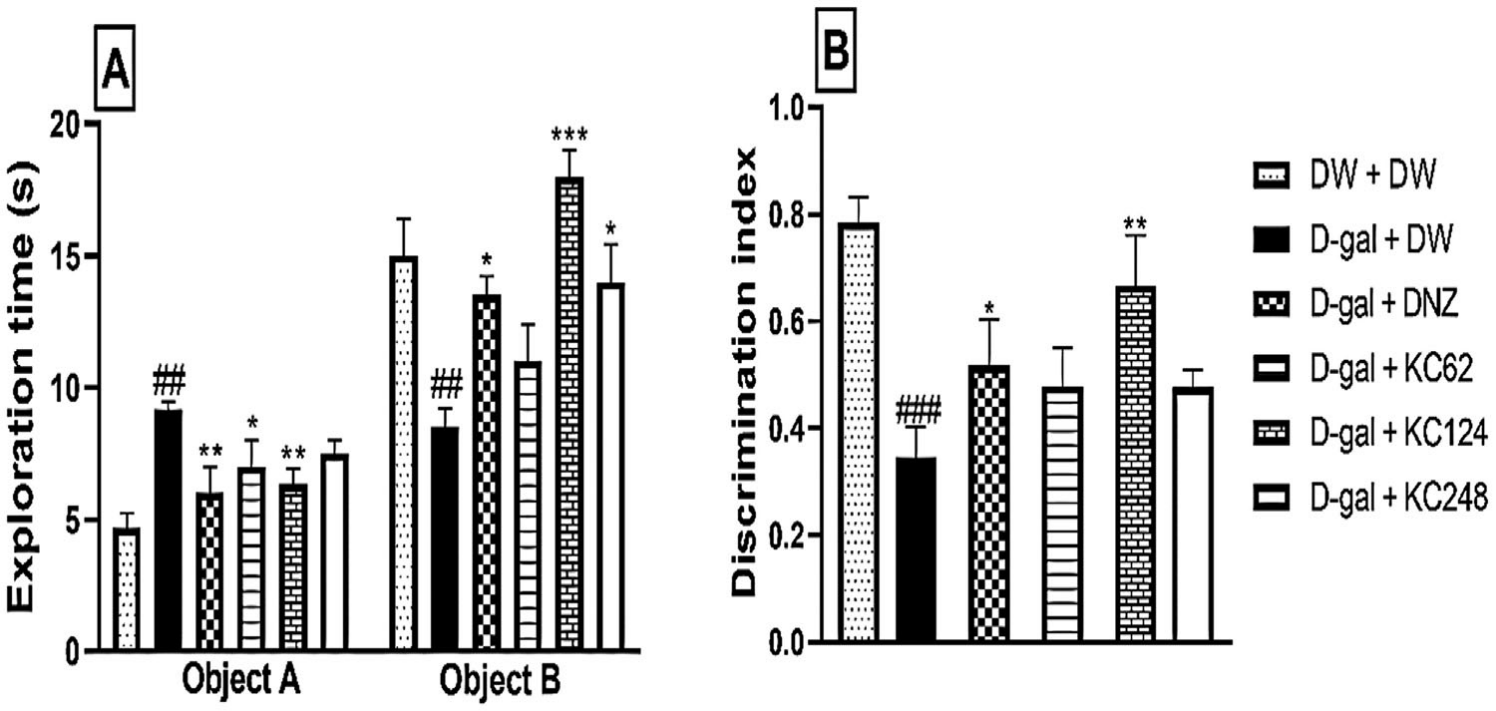
Effect of an aqueous extract of *K. crenata* on the exploration time (A) and the discrimination index (B). Each bar represents the mean ± SEM; *N* = 8. D‐gal + DNZ, positive control group; D‐gal + DW, negative control group; D‐gal + KC (62, 124, and 248), test groups treated with the extract at respective doses of 62, 124, and 248 mg/kg; D‐gal, D‐galactose (350 mg/kg); DNZ, donepezil (2 mg/kg); DW + DW, control group; DW, distilled water (10 mL/kg); KC (62, 124, and 248), aqueous extract of *K. crenata* at the respective doses of 62, 124, and 248 mg/kg; Object A, old object; Object B, new object. ##*p* < 0.01, ###*p* < 0.001 compared to the control group (DW + DW); *p < 0.05, **p < 0.01, ***p < 0.001 compared to the negative control group (D‐gal + DW)

In the negative control group, D‐galactose (D‐gal) increased the time to explore object A (9.17 ± 0.16 s, *p* < 0.01) when compared to the control group (Figure 3A). The extract at the doses of 62 and 124 mg/kg reduced this time to 7.50 ± 0.50 s (*p* < 0.05) and 6.33 ± 0.59 s (*p* < 0.01), respectively. Donepezil reduced this time to 6.00 ± 0.57 s (*p* < 0.01).

In the negative control group, D‐gal reduced the time to explore object B (8.50 ± 0.50 s, *p* < 0.01) compared to the control group (Figure 3A). The extract at the doses of 124 and 248 mg/kg increased this time to 18.00 ± 1.70 s (*p* < 0.001) and 14.02 ± 1.41 s (*p* < 0.05), respectively. Donepezil also increased this time to 13.50 ± 0.50 s (*p* < 0.05).

In the negative control group, D‐gal reduced the DI (0.34 ± 0.03, *p* < 0.001) compared to the control group (Figure 3B). The extract at a dose of 124 mg/kg markedly increased this index to 0.66 ± 0.60 (*p* < 0.01). Donepezil also increased this index to 0.47 ± 0.01 (*p* < 0.05).

### 3.2 Effect of an Aqueous Extract of *K. crenata* on Spatial Memory During the Acquisition Phase

On Days 1, 2, and 3, administration of the D‐gal did not affect the latency to reach the platform in the negative control group compared to the control group (Table 1). This also applies to the extract and donepezil. On Day 4, this time increased to 27.36 ± 7.54 s (*p* < 0.001) in the negative control group compared to the control group (Table 1). However, the extract significantly decreased this time at all doses, especially at the dose of 124 mg/kg where this time was remarkably shortened (7.75 ± 1.51 s, *p* < 0.001). Donepezil also reduced this parameter to 10.77 ± 2.79 s (*p* < 0.01) (Table 1).

**TABLE 1:** Effect of an aqueous extract of *K. crenata* on the latency to reach the platform during the acquisition phase.

Treatment	Day 1 lat. (s)	Day 2 lat. (s)	Day 3 lat. (s)	Day 4 lat. (s)
DW + DW	18.53 ± 3.94	13.77 ± 2.26	16.62 ± 2.13	7.71 ± 1.30
D‐gal + DW	31.74 ± 6.56	23.06 ± 8.91	25.28 ± 4.89	27.36 ± 7.54^###^
D‐gal + DNZ	22.86 ± 4.65	13.71 ± 2.70	14.45 ± 2.53	10.77 ± 2.79^**^
D‐gal + KC62	21.68 ± 3.45	12.08 ± 2.22	14.12 ± 2.26	15.01 ± 1.61^**^
D‐gal + KC124	20.82 ± 2.43	12.86 ± 2.55	13.69 ± 3.70	7.75 ± 1.51^***^
D‐gal + KC248	29.07 ± 9.70	21.66 ± 3.93	18.21 ± 4.19	14.61 ± 3.43^**^

*Note*: Each value represents the mean ± SEM. *NN* = 8,

Abbreviations: D‐gal + DNZ: Positive control group; D‐gal + DW: Negative control group; D‐gal + KC (62, 124, and 248): Test groups treated with the extract at doses of 62, 124, and 248 mg/kg, respectively; D‐gal: D‐galactose (350 mg/kg); DNZ: Donepezil (2 mg/kg); DW + DW: control group; DW: Distilled water (10 mL/kg); KC (62, 124, and 248): Aqueous extract of *K. crenata* at doses of 62, 124, and 248 mg/kg, respectively; Lat.: latency.

###*p* < 0.001 compared to the control group (DW + DW); *p < 0.05, **p < 0.01, ***p < 0.001 compared to the negative control group (D‐gal + DW).

### 3.3 Effect of an Aqueous Extract of *K. crenata* on Spatial Memory During the Retention Phase

**FIGURE 4:**
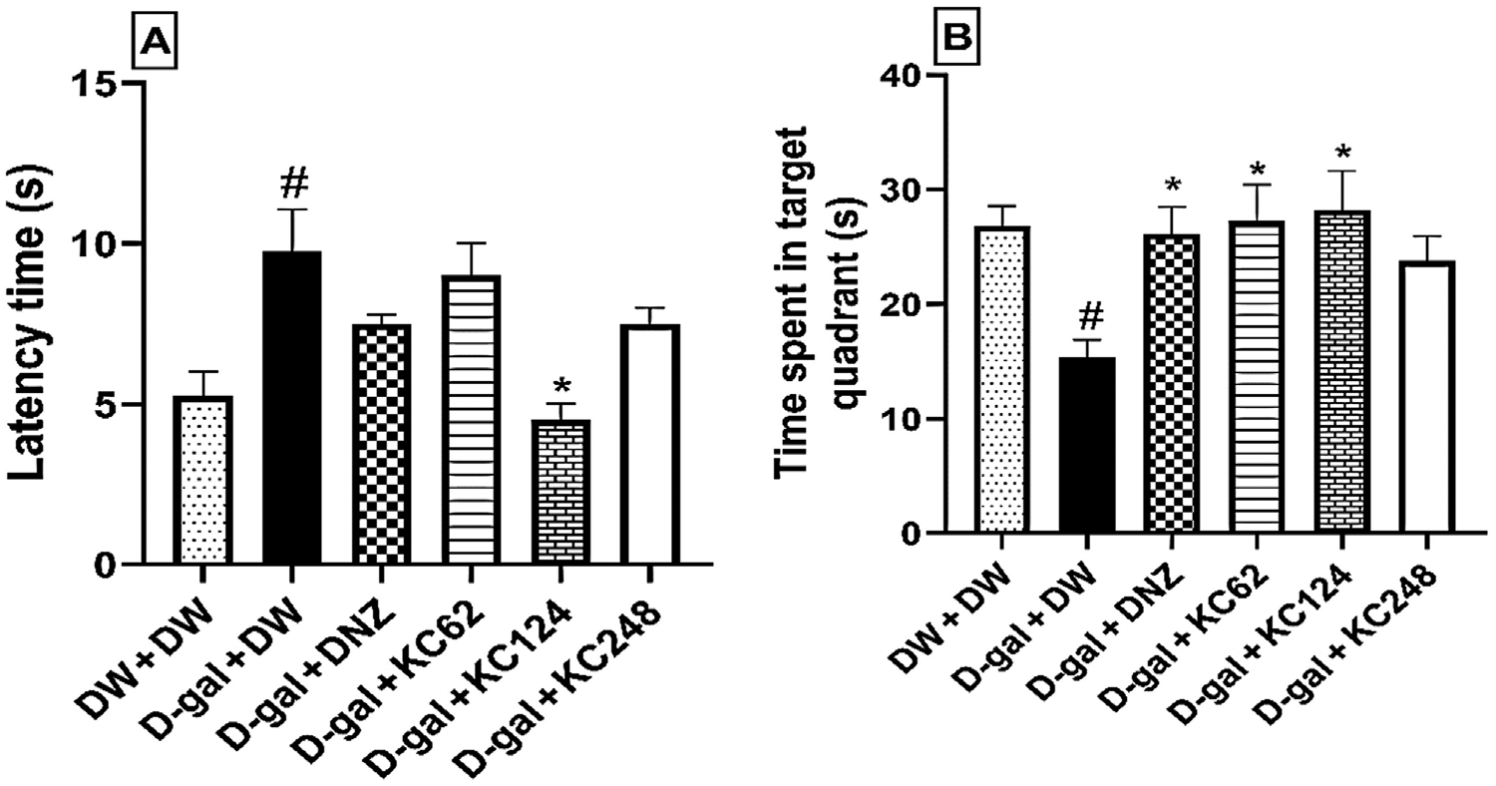
Effect of an aqueous extract of *K. crenata* on the latency to reach the target quadrant (A) and the time spent in the target quadrant (B) during the retention phase. Each bar represents the mean ± SEM; *N* = 8; ##*p* < 0.01, ###*p* < 0.001 compared to the control group (DW + DW). D‐gal + DNZ, positive control group; D‐gal + DW, negative control group; D‐gal + KC (62, 124, and 248), test groups treated with the extract at doses of 62, 124, and 248 mg/kg, respectively; D‐gal, D‐galactose (350 mg/kg); DNZ, donepezil (2 mg/kg); DW + DW, control group; DW, distilled water (10 mL/kg); KC (62, 124, and 248), aqueous extract of *K. crenata* at doses of 62, 124, and 248 mg/kg, respectively. #*p* < 0.05 compared to the control group (DW + DW); *p < 0.05 compared to the negative control group (D‐gal + DW).

In the negative control group, D‐gal increased the time (9.75 ± 0.75 s, *p* < 0.05) to reach the target quadrant compared to the control group (Figure 4A). The extract (124 mg/kg) markedly reduced this time to 4.50 ± 0.50 s (*p* < 0.05) (Figure 4A).

Regarding the time spent in the target quadrant (Figure 4B), D‐gal reduced this time to 15.40 ± 1.50 s (*p* < 0.05) in the negative control group compared to the control group. The extract at the doses of 62 and 124 mg/kg increased this time to 27.40 ± 3.17 s (*p* < 0.05) and 28.20 ± 3.44  s (*p* < 0.05), respectively. Similar results were obtained with donepezil, which increased this time to 26.20 ± 2.33 s (*p* < 0.05) (Figure 4B).

### 3.4 Effect of an Aqueous Extract of *K. crenata* on Anxiety‐Like Behavior

Table 2 shows that the number of groomings in the negative control group decreased to 8.00 ± 1.15 (*p* < 0.001) compared to the control group. The extract (124 mg/kg) and donepezil remarkably increased this number to 20.00 ± 0.50 (*p* < 0.001) and 17.50 ± 0.50 (*p* < 0.001), respectively.

**TABLE 2:** Effect of an aqueous extract of *K. crenata* on anxiety‐like behavior.

	Number of groomings	Number of crossings	Time spent in the center (s)	Number of stools	Number of rearings
DW+DW D‐gal+DW D‐gal+DNZ D‐gal+KC62 D‐gal+KC124 D‐gal+ KC 248	16.00 ± 1.00 8.00 ± 1.15## 17.5 ± 0.50*** 12.66 ± 0.88* 20.00 ± 0.50*** 17.00 ±0.50***	24.14 ± 2.13 15.40 ± 1.50 26.16 ± 2.33* 28.83 ± 2.26** 28.20 ± 3.44 23.80 ± 2.15*	15.00 ± 1.15 7.00 ± 1.15 12.33 ± 1.45 18.00 ± 3.00 * 15.33 ± 2.60 22.50 ± 2.50 **	2.00 ± 1.00 6.66 ± 0.33## 1.66 ± 0.33** 2.75 ± 0.47** 1.25 ± 0.75*** 3.00 ± 1.00*	7.00 ± 1.68 21.50 ± 0.50### 9.66 ± 0.64*** 15.50 ± 0.50 9.66 ± 0.12** 12.50 ± 2.50*

*Note*: Each value represents the mean ± SEM. *N* = 8,

Abbreviations: D‐gal + DNZ: positive control group; D‐gal + DW: negative control group; D‐gal + KC (62, 124, and 248): test groups treated with the extract at doses of 62, 124, and 248 mg/kg, respectively; D‐gal: D‐galactose (350 mg/kg); DNZ: donepezil (2 mg/kg); DW + DW: control group; DW: distilled water (10 mL/kg); KC (62, 124, and 248): aqueous extract of *K. crenata* at doses of 62, 124, and 248 mg/kg, respectively; Lat.: latency.

##*p* < 0.01, ###*p* < 0.001 compared to the control group (DW + DW); *p < 0.05, **p < 0.01, ***p < 0.001 compared to the negative control group (D‐gal + DW).

*N*

The number of crossings increased from 24.14 ± 2.13 in the control group to 15.40 ± 1.50 in the negative control group (Table 2). The extract (62 and 248 mg/kg) increased this number to 28.80 ± 2.26 (*p* < 0.01) and 23.80 ± 2.15 (*p* < 0.05), respectively. Donepezil also increased this number to 26.20 ± 2.33 (*p* < 0.05).

The time spent in the center of the open field decreased from 15.00 ± 1.15 s in the control group to 7.00 ± 1.15 s in the negative control group (Table 2). The extract (62 and 248 mg/kg) increased this time to 18.00 ± 1.30 s (*p* < 0.05) and 22.50 ± 2.50  s (*p* < 0.01), respectively. Donepezil increased this time to 12.33 ± 1.45 s (*p* < 0.05).

The number of stools produced by the negative control group increased to 6.66 ± 0.33 (*p* < 0.01) compared to the control group (Table 2). The extract at all doses reduced this number. However, the extract at a dose of 124 mg/kg markedly reduced this number to 1.25 ± 0.75 (*p* < 0.001). Donepezil also decreased this number to 1.66 ± 0.33 (*p* < 0.01).

The number of rearings increased from 7.00 ± 1.68 in the control group to 21.5 ± 0.50 (*p* < 0.01) in the negative control group (Table 2). The extract at the doses of 124 and 248 mg/kg reduced this number to 9.66 ± 0.12 (*p* < 0.01) and 12.50 ± 2.50 (*p* < 0.05), respectively. Donepezil also reduced this parameter to 5.50 ± 0.64 (*p* < 0.001).

### 3.5 Effect of an Aqueous Extract of *K. crenata* on Acetylcholinesterase and Butyrylcholinesterase Activity in the Hippocampus

**FIGURE 5:**
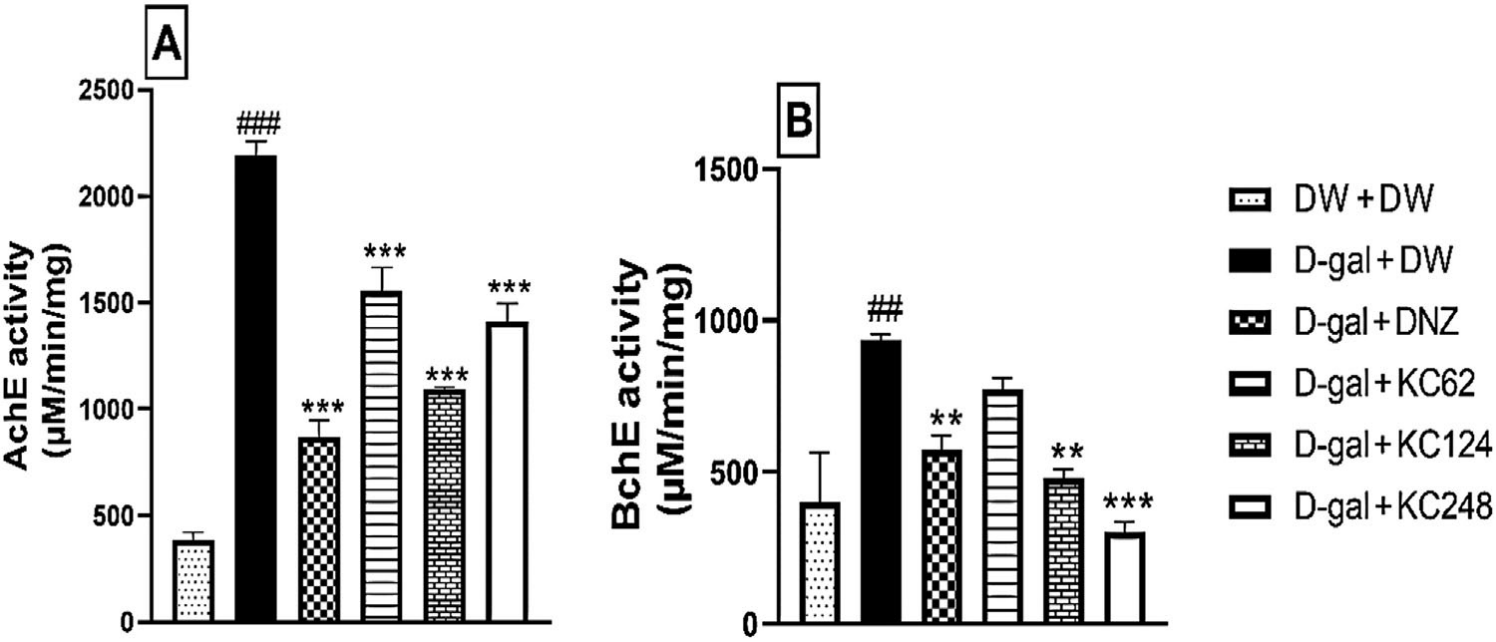
Effect of an aqueous extract of *K. crenata* on the activity of acetylcholinesterase (A) and butyrylcholinesterase (B) in the hippocampus. Each bar represents the mean ± SEM; *N* = 8. AchE, acetylcholinesterase; BchE, butyrylcholinesterase; D‐gal + DNZ, positive control group; D‐gal + DW, negative control group; D‐gal + KC (62, 124, and 248), test groups treated with the extract at doses of 62, 124, and 248 mg/kg, respectively; D‐gal, D‐galactose (350 mg/kg); DNZ, donepezil (2 mg/kg); DW + DW, control group; DW, distilled water (10 mL/kg); KC (62, 124, and 248), aqueous extract of *K. crenata* at doses of 62, 124, and 248 mg/kg, respectively. ##*p* < 0.01, ###*p* < 0.001 compared to the control group (DW + DW); **p < 0.01, ***p < 0.001 compared to the negative control group (D‐gal + DW).

Analysis of Figure 5A shows an increase in AchE activity from 385.35 ± 2.62 µM/min/mg in the control group to 2190.03 ± 1.28 µM/min/mg (*p* < 0.001) in the negative control group. The extract (64, 124, and 248 mg/kg) decreased this activity to 1553.01 ± 7.11 (*p* < 0.001), 1097.50 ± 5.19 (*p* < 0.001), and 1412.70 ± 5.13 µM /min/mg (*p* < 0.001), respectively. Similarly, donepezil reduced this activity to 869.23 ± 5.52 µM/min/mg (*p* < 0.001) (Figure 5A).

In Figure 5B, D‐gal increased BchE activity to 937.27 ± 1.28 µM/min/mg (*p* < 0.01) in the negative control group compared to the control group. The extract (124 and 248 mg/kg) decreased this activity to 482.53 ± 18.30 (*p* < 0.01) and 302.80 ± 23.50 (*p* < 0.001) µM/min/mg, respectively. Donepezil also decreased this activity to 574.01 ± 2.76 µM/min/mg (*p* < 0.01) (Figure 5B).

### 3.6 Effect of an Aqueous Extract of *K. crenata* on Oxidative Stress in the Hippocampus

**FIGURE 6:**
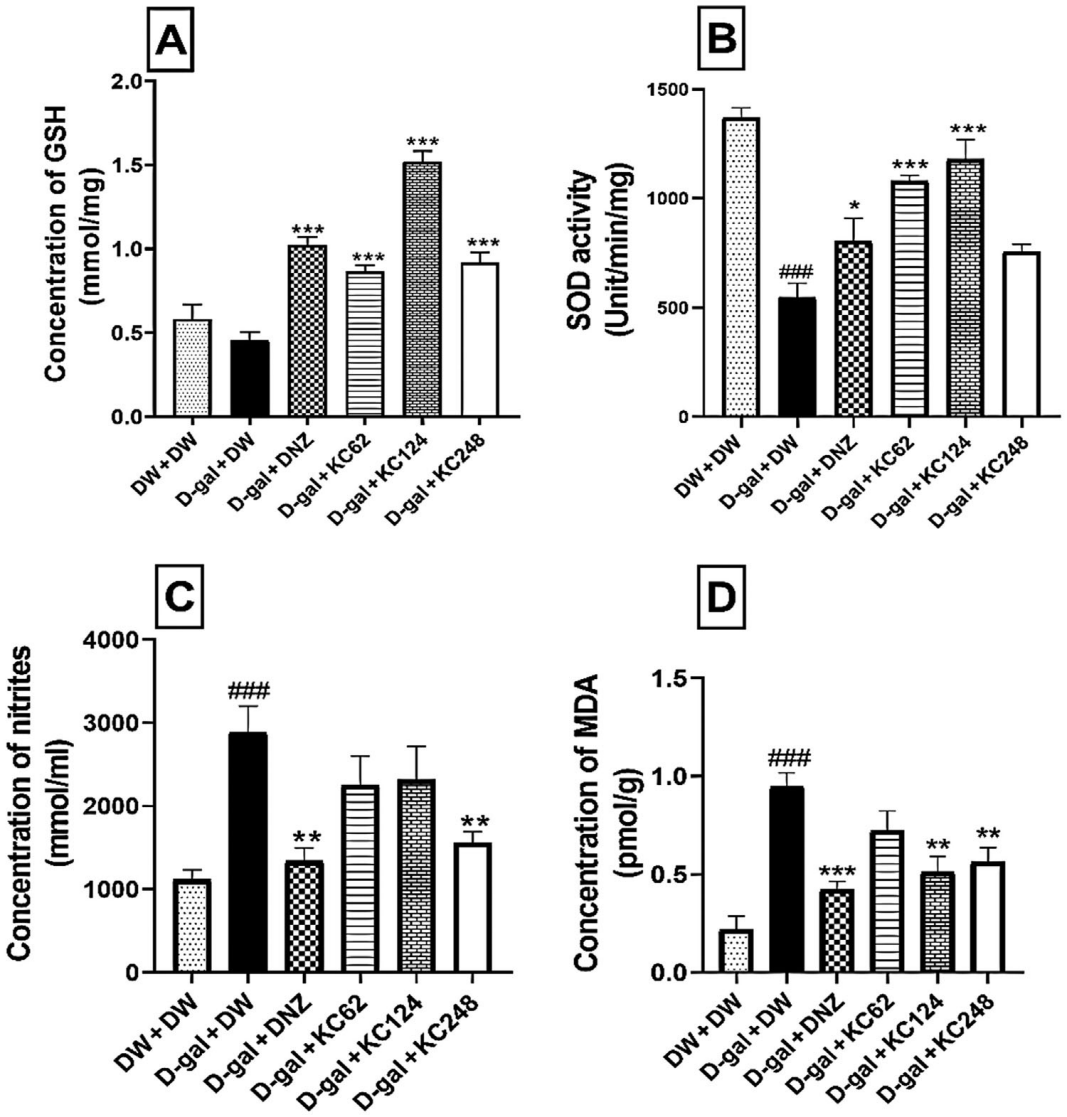
Effect of an aqueous extract of *K. crenata* on GSH concentration (A), SOD activity (B), nitrite concentration (C), and MDA concentration (D) in the hippocampus. Each bar represents the mean ± SEM; *N* = 8. D‐gal + DNZ, positive control group; D‐gal + DW, negative control group; D‐gal + KC (62, 124, and 248), test groups treated with the extract at doses of 62, 124, and 248 mg/kg, respectively; D‐gal, D‐galactose (350 mg/kg); DNZ, donepezil (2 mg/kg); DW + DW, control group; DW, distilled water (10 mL/kg); GSH, Reduced glutathione; KC (62, 124, and 248), aqueous extract of *K. crenata* at doses of 62, 124, and 248 mg/kg respectively; MDA, malondialdehyde; SOD, superoxide dismutase. ###*p* < 0.001 compared to the control group (DW + DW); *p < 0.05, **p < 0.01, ***p < 0.001 compared to the negative control (D‐gal + DW).

The GSH concentration decreased to 0.45 ± 0.03 mmol/mg in the negative control group compared to the control group (Figure 6A). The extract at all doses increased this concentration. However, the extract at the highest dose increased this concentration to 1.52 ± 0.04 mmol/mg (*p* < 0.001). Donepezil also increased this concentration to 1.02 ± 0.03 mmol/mg (*p* < 0.001) (Figure 6A).

The SOD activity increased to 548.32 ± 1.93 units/min/mg (*p* < 0.001) in the negative control compared to the control group (Figure 6B). The extract at the doses of 62 and 124 mg/kg reduced this activity to 1082.51 ± 1.69 (*p* < 0.001) and 1180.23 ± 7.33 units/min/mg (*p* < 0.001), respectively. Donepezil also decreased this activity to 806.00 ± 2.09 units/min/mg (*p* < 0.001) (Figure 6B).

The nitrite concentration increased to 2890.02 ± 1.30 mmol/mL in the negative control compared to the control group (Figure 6C). The extract at the dose of 248 mg/kg decreased this concentration to 1556.00 ± 7.08 mmol/mL (*p* < 0.01). Donepezil also reduced this concentration by 1342.02 ± 1.08 mmol/mL (*p* < 0.001) (Figure 6C).

The MDA concentration increased to 0.95 ± 0.04 pmol/g in the negative control group compared to the control group (Figure 6D). The extract at the doses of 124 and 248 mg/kg reduced this concentration to 0.51 ± 0.03 (*p* < 0.01) and 0.56 ± 0.04 pmol/g (*p* < 0.01), respectively. Likewise, donepezil decreased this concentration to 0.42 ± 0.02 pmol/g (*p* < 0.001) (Figure 6D).

### 3.7 Effect of an Aqueous Extract of *K. crenata* on the Concentration of Some Proinflammatory Markers in the Hippocampus and Blood

**FIGURE 7:**
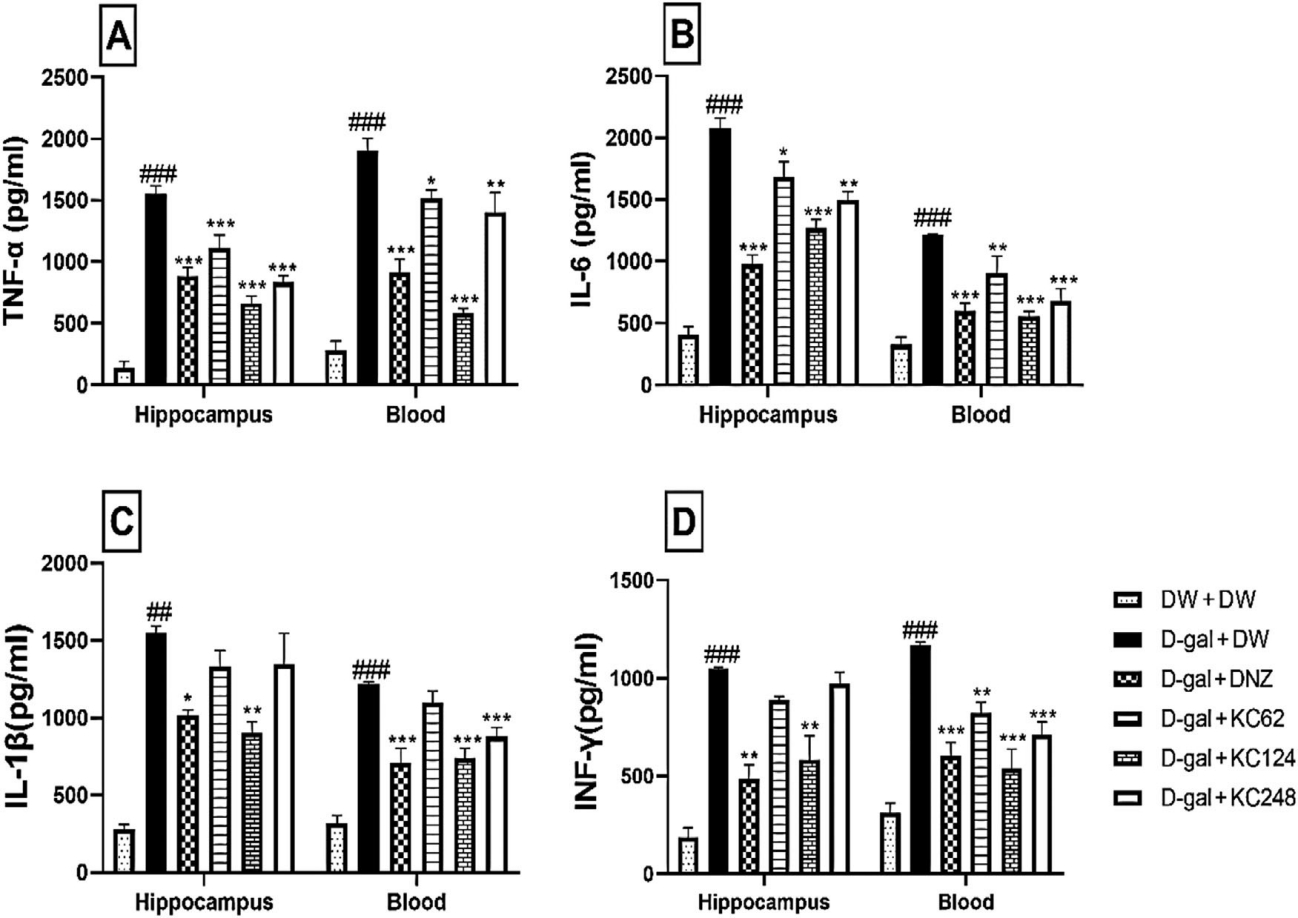
Effect of an aqueous extract of *K. crenata* on TNF‐α concentration (A), IL‐6 concentration (B), IL‐1β concentration (C), and IFN‐γ concentration (D) in the hippocampus and blood. Each bar represents the mean ± SEM; *N* = 8. D‐gal + DNZ, positive control group; D‐gal + DW, negative control group; D‐gal + KC (62, 124, and 248), test groups treated with the extract at doses of 62, 124, and 248 mg/kg, respectively; D‐gal, D‐galactose (350 mg/kg); DNZ, donepezil (2 mg/kg); DW + DW, control group; DW, distilled water (10 mL/kg); IFN‐γ, interferon gamma; IL‐1β, interleukin‐1 beta; IL‐6, interleukin‐6; KC (62, 124, and 248), aqueous extract of *K. crenata* at doses of 62, 124, and 248 mg/kg, respectively; TNF‐α, tumor necrosis factor alpha. ##*p* < 0.01, ###*p* < 0.001 compared to the control group (DW + DW); *p < 0.05, **p < 0.01, ***p < 0.001 compared to the negative control group (D‐gal + DW).

In the negative control group, D‐gal increased the concentration of TNF‐α to 1551.75 ± 3.02 pg/m (*p* < 0.001) in the hippocampus and 1904.80 ± 7.80 pg/mL (*p* < 0.001) in the blood compared to the control group (Figure 7A). All doses of the extract lowered TNF‐α concentration. The extract (62 mg/kg) markedly reduced this concentration to 663.87 ± 8.70 pg/mL (*p* < 0.001) in the hippocampus. In the blood, all doses of the extract reduced the TNF‐α concentration, with a pronounced effect at a dose of 124 mg/kg (586.12 ± 2.30 pg/mL, *p* < 0.001). Donepezil also reduced the concentration of TNF‐α in the hippocampus (883.04 pg/mL, *p* < 0.001) and blood (917.15 ± 2.60 pg/mL, *p* < 0.001) (Figure 7A).

IL‐6 concentration in the negative control group increased in the hippocampus (2078.82 ± 2.32 pg/mL, *p* < 0.001) and blood (1213.52 ± 3.20 pg/mL, *p* < 0.001) compared to the control group (Figure 7B). The extract at the highest dose reduced this concentration to 1266.00 ± 5.60 ρg/mL (*p* < 0.001) in the hippocampus. In the blood, the extract decreased this concentration at all doses. However, the extract at doses of 124 and 248 mg/kg reduced this concentration to 552.15 ± 3.60 (*p* < 0.001) and 678.56 ± 8.02 pg/mL (*p* < 0.001), respectively. Donepezil also reduced this concentration to 973.58 ± 3.20 pg/mL (*p* < 0.001) in the hippocampus and 1213.47 ± 8.90 pg/mL(*p* < 0.001) in the blood (Figure 7B).

In the negative control group, D‐gal increased IL‐1β concentration to 1550.23 ± 8.70 (*p* < 0.001) in the hippocampus and 1216.47 ± 8.90 pg/mL (*p* < 0.001) in the blood compared to the control group (Figure 7C). The extract (124 mg/kg) markedly decreased this concentration to 903.02 ± 5.05 pg/mL (*p* < 0.01) in the hippocampus and 845.12 ± 2.31 pg/mL (*p* < 0.001) in the blood. Likewise, donepezil reduced IL‐1β concentration in the hippocampus (*p* < 0.05) and blood (*p* < 0.001) (Figure 7C).

In the negative control group, the concentration of IFN‐γ increased to 1047.69 ± 8.70 ρg/mL (*p* < 0.001) in the hippocampus and 1171.54 ± 1.00 pg/mL (*p* < 0.001) in the blood compared to the control group (Figure 7D). The extract at a dose of 124 mg/kg reduced this concentration to 583.58 ± 5.40 pg/mL (*p* < 0.01) in the hippocampus. In the blood, the extract (124 and 248 mg/kg) decreased this concentration to 539.7 ± 3.20 (*p* < 0.001) and 711.38 ± 7.90 pg/mL (*p* < 0.001), respectively. Donepezil also reduced this concentration to 484.7 ± 8.30 pg/mL (*p* < 0.01) in the hippocampus and 604.45 ± 8.80 pg/mL (*p* < 0.01) in the blood (Figure 7D).

### 3.8 Effect of an Aqueous Extract of *K. crenata* on Neuronal Alterations in the Hippocampus

**FIGURE 8:**
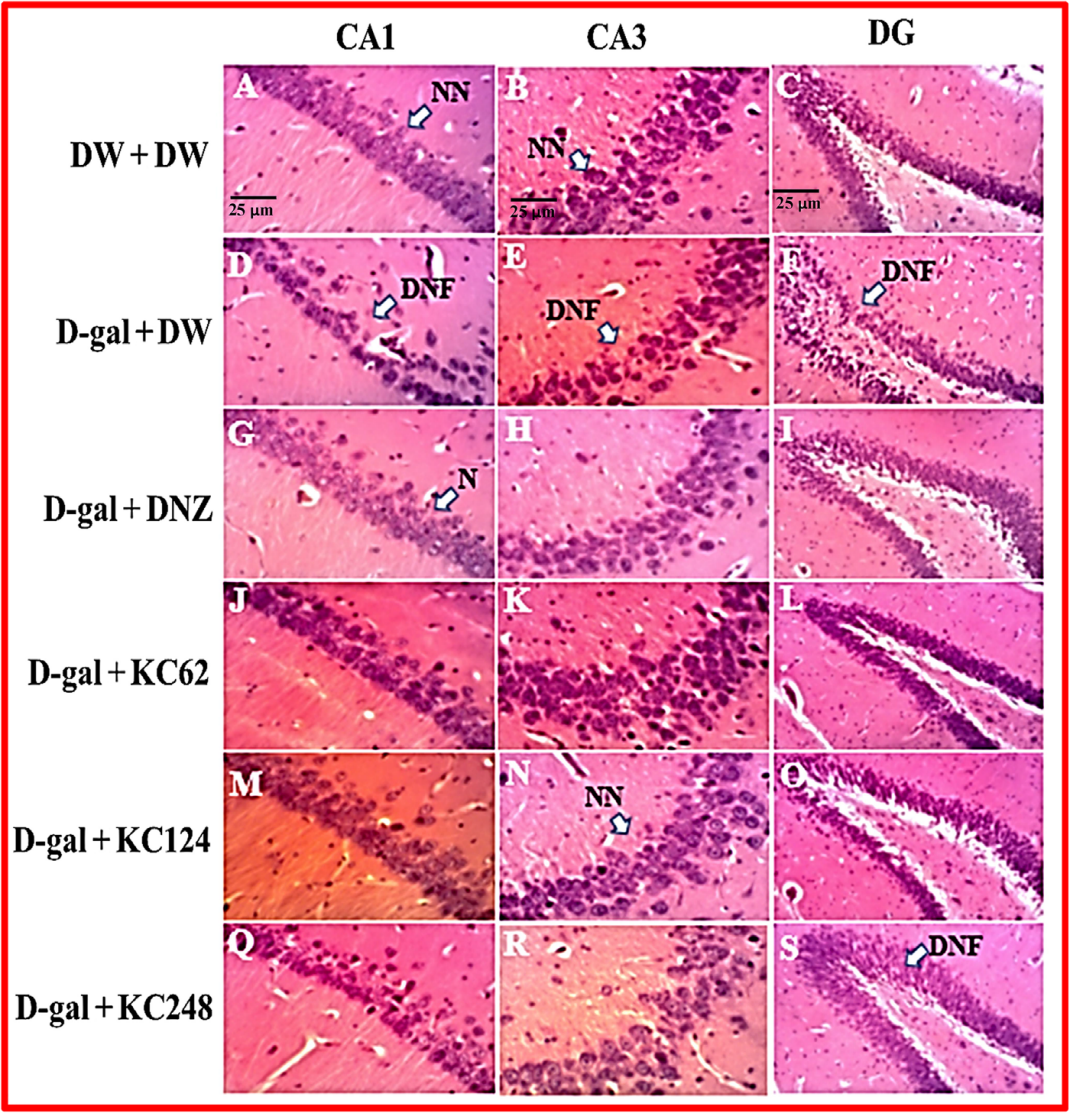
Photomicrographs of CA1 and CA3 layers (250X) and dentate gyrus (100X) after hematoxylin‐eosin staining (scale = 25 µm). CA1,3, Ammon's horns 1 and 3; DG, Dentate gyrus; D‐gal + DNZ, Positive control group; D‐gal + DW, Negative control group; D‐gal + KC (62, 124, and 248), Test groups treated with the extract at doses of 62, 124, and 248 mg/kg, respectively; D‐gal, D‐galactose (350 mg/kg); DNF, Neurofibrillary degeneration; DNZ, Donepezil (2 mg/kg); DW + DW, control group; DW, Distilled water (10 mL/kg); KC (62, 124, and 248), Aqueous extract of *K. crenata* at doses of 62, 124, and 248 mg/kg, respectively; NN, Normal neuron.

The microarchitecture of the hippocampus of the control group shows intact neurons in the CA1 and CA3 layers (Figure 6A and B) and regular thickness of the dentate gyrus (Figure 8A–C). In the negative control group, several pathological modifications were observed such as a decrease in the number of alive neurons, cell disorganization, and neuronal cytolysis in the hippocampus (Figure 8D–F). Interestingly, the animals treated with the extract (62 and 124 mg/kg) and donepezil showed a similar hippocampal structure to the control group (Figure 8G–I).

In addition, Table 3 shows the effect of *K. crenata* on the density of neurons in the CA1 and CA3 layers. In the negative control group, D‐gal decreased the number of neurons to 38.45% (*p* < 0.001) in the CA1 layer and 38.51% (*p* < 0.05) in the CA3 layer compared to the control group. The extract (62 and 124 mg/kg) increased this number to 48.76% (*p* < 0.001) and 46.61% (*p* < 0.001) in the CA1 layer, respectively. These doses equally increased this number to 50.58% (*p* < 0.05) and 51.85% (*p* < 0.05) in the CA3 layer, respectively. Donepezil increased the number of neurons to 45.83% (*p* < 0.001) in the CA1 layer and 44.13% (*p* < 0.05) in the CA3 layer (Table 3).

**TABLE 3:** Effect of an aqueous extract of *K. crenata* on the density of neurons in CA1 and CA3 layers.

	DW+DW	D‐gal+DW	D‐gal+DNZ	D‐gal+KC62	D‐gal+KC124	D‐gal+KC248
CA1 (n/µm^2^)	98.30 ± 0.88	60.50 ± 1.30**^###^**	87.30 ± 3.93^***^	90.00 ± 2.89^***^	88.70 ± 3.84^***^	63.00 ± 2.65
CA3 (n/µm^2^)	52.70 ± 1.60	32.40 ± 1.50**^#^**	45.30 ± 0.31^*^	48.79 ± 1.72^*^	49.20 ± 1.97^*^	44.30 ± 0.67

*Note*: Each value represents the mean ± SEM. *N* = 8,

Abbreviations: D‐gal + DNZ: Positive control group; D‐gal + DW: Negative control group; D‐gal + KC (62, 124, and 248): Test groups treated with the extract at doses of 62, 124, and 248 mg/kg, respectively; D‐gal: D‐galactose (350 mg/kg); DNZ: Donepezil (2 mg/kg); DW + DW: control group; DW: Distilled water (10 mL/kg); KC (62, 124, and 248): Aqueous extract of *K. crenata* at doses of 62, 124, and 248 mg/kg, respectively; Lat.: latency.

##*p* < 0.01, ###*p* < 0.001 compared to the control group (DW + DW); *p < 0.05, **p < 0.01, ***p < 0.001 compared to the negative control group (D‐gal + DW).

*N*

### 3.9 Quantitative Phytochemical Analysis

Compared to standards, total phenolic compounds (98.03 ± 0.32 mg GAE/g), total alkaloids (31.24 ± 0.29%), and flavonoids contents (29.17 ± 0.53 mg RE/g) were abundant while condensed tannins (3.16 ± 0.19 mg Cat E/g) and saponins (5.77 ± 0.42%) were less common (Table 4).

**TABLE 4:** Quantitative analysis of an aqueous extract of *K. crenata*.

Chemical compounds	Unit	Mean
Alkaloids	%	31.24 ± 0.29
Flavonoids	mg RE/g	29.17 ± 0.53
Tannins	mg CatE/g	3.16 ± 0.19
Polyphenols	mg GAE/g	98.03 ± 0.32
Saponins	%	5.77 ± 0.42

Abbreviations: %: percentage; CatE: catechin equivalent; GAE: gallic acid equivalent; RE: rutin equivalent.

## 4 Discussion

The present study aimed to evaluate the properties of *K. crenata* extract on D‐galactose‐induced amnesia, oxidative stress, and inflammation in rats. The administration of D‐galactose (D‐gal) increased the recognition of the ancient object over the novel. The inability of animals to quickly recall an object they have previously been exposed reflects an impairment of short‐term memory (Sadigh‐Eteghad et al. 2015). D‐gal can cause cognitive impairment by inducing aging, oxidative stress, and inflammation. In addition, D‐gal induces neuronal degeneration and low levels of Ach in the brain. *K. crenata* increased the index of discrimination and recognition of the new object. These data indicate that the extract possesses an anti‐amnesic effect against working memory deficits. Secondary metabolites such as polyphenols and flavonoids have been shown to reduce such behavioral alterations in rats (Enrique 2017). Kalanchoe species contain high amounts of these metabolites (Patwekar et al. 2010). Hence, these chemicals could partially explain the anti‐amnesic activity of the extract (Asiedu‐Gyekye et al. 2013).

Patients with AD complaint about long‐term memory impairment. The effect of the extract on long‐term spatial memory was therefore assessed in the Morris water maze. Not surprisingly, our findings revealed that D‐gal administration caused an increase in latency to reach the target quadrant. D‐gal is an antagonist of muscarinic acetylcholine (Ach) receptors, with Ach being the major neurotransmitter involved in the memory process (Li et al. 2021; Oke and Tracey 2008). In this study, rats treated with *K. crenata* extract spent more time in this quadrant compared to the negative control group. Our results suggest that *K. crenata* extract may improve learning and reference memory (long‐term memory). The extract protected the brain from neuronal degeneration in the CA1 and CA3 layers. These regions regulate the memory process, particularly in the generation of long‐term memory (Xiao et al. 2011). The CA3 region is associated with coding, while the CA1 region plays a role in acquisition and consolidation (Daumas et al. 2005). The extract could therefore improve the memory process by protecting the brain from neuronal loss. All this indicates the functional and morphological protective properties of *K. crenata* extract.

Evaluation of the exploratory and emotional activities revealed that the extract increased the number of crossings, the number of groomings, and the time in the center of the device. The change in these parameters indicates improved motricity and exploration in rats. Increased locomotion expresses a reduction in anxiety and stress (Ngo Bum et al. 2011). With this in mind, the antiamnesic effect of the extract is not the result of anxiety and stress experienced by rats, but it is rather the result of the stimulation of brain areas that control memory. Indeed, environmental and water stress is likely to introduce biases leading to false positive results. Thus, the ability of the extract to neutralize these factors increases the internal validity of the study.

During AD, deposition of Aβ induces overactivity of AchE and Ach(Kandeda et al., 2025). AchE metabolizes acetylcholine, leading to memory impairment and neuronal dysfunction (Kandeda et al., 2025). Administration of D‐gal increased AchE and BchE activity, and consequently, a decrease in Ach concentration (Shwe et al. 2018). Our study reveals that the aqueous extract decreased the activity of these enzymes. This inhibitory activity suggests that the extract protects cholinergic neurons from D‐gal‐induced damage. The neuroprotective potential of the extract is also confirmed here. Furthermore, the study by Rola (Milad, El‐Ahmady, and Singab 2014) has shown that Kalanchoe species are endowed with anticholinesterase activity, which may be due to chemicals (secondary metabolites) that interact with cholinergic neurotransmission (Milad, El‐Ahmady, and Singab 2014). The phytochemical analysis of *K. crenata* revealed a huge amount of tannins, terpenes, alkaloids, flavonoids, polyphenols, saponins, and tannins. These compounds have been shown to inhibit some kinases (PKC, PI3K, and tyrosine kinase) that are involved in the cholinergic system (Rebas et al., 2020). Further research in this direction will help to understand how extract components interact with the cholinergic circuit.

AD is also characterized by the production of free radicals and reactive oxygen species (ROS) (Rita Cardoso et al. 2014). In this study, D‐gal induced oxidative stress through an increase in MDA and nitrite levels, and through a decrease in GSH and SOD levels. In the hippocampus, D‐gal is converted into galactitol, which accumulates in cells, causing osmotic stress and increasing ROS production (Socci, Crandall, and Arendash 1995). ROS causes membrane damage and cellular death through three main mechanisms including lipid peroxidation, protein oxidation, and DNA damage (Rita Cardoso et al. 2014). Several studies reported that compounds with antioxidant activity could inhibit the deposition of Aβ in the brain and thus improve cognition (Brot and Weissbach 2000). In this study, *K. crenata* extract mitigates oxidative stress by reducing MDA level and increasing those of GSH and SOD. The antioxidant activities of the extract are likely linked to free radical reuptake (Kandeda, Mabou, and Moutchida 2022; Kandeda et al. 2021; Kandeda, Moto et al. 2021). This activity could be due to the presence of some secondary metabolites identified in the extract such as flavonoids, tannins, and phenols. In fact, tannins, phenols, alkaloids, and flavonoids are free radical scavengers (Cheignon et al. 2018). Our results also agree with those of Asiedu‐Gyekye et al. (2013) who demonstrated antioxidant and anti‐ischemic effects of the methanolic extract of *K. crenata*.

It is known that neuro‐inflammation increases the severity of AD through Aβ deposition and tau protein phosphorylation (Rogers et al. 1988). In AD, Aβ aggregates are a major driver of microglial cell activation. Indeed, these molecules can be phagocytosed by microglia. This can trigger an inflammatory response. Chronically, activated microglia release numerous proinflammatory molecules, including IL‐1β, IL‐6, TNF‐α, and IFN‐γ (Cheignon et al. 2018). In the negative control group, D‐gal induced an increase in the levels of proinflammatory cytokines (IL‐1β, IL‐6, TNF‐α, and IFN‐γ) compared to the control group. Administration of D‐gal is known to stimulate the nuclear factor kappa‐light‐chain enhancer of B cells (NF‐kB) (Shwe et al. 2018). This leads to cytokine production. The present research revealed that *K. crenata* extract reduced the concentration of cytokines in the hippocampus and blood, confirming its anti‐inflammatory properties documented in the literature. These results are consistent with those of Varma et al. (1979) who demonstrated the anti‐inflammatory effects of *K. crenata*. In addition, the flavonoids in the extract inhibited proinflammatory cytokine production and NF‐kB activation (Rathee et al. 2009). The attenuation of proinflammatory cytokines in the hippocampus may explain the neuroprotective effect of the *K. crenata* extract (Kandeda, Menvouta et al. 2022; Kandeda, Nguedia et al. 2021; Kandeda, Nguedia et al. 2022; Kandeda, Nodeina, and Mabou 2022). Further studies are needed to unravel all the mechanisms involved in the anti‐inflammatory activity of the extract.

## 5 Conclusion

This study aimed to investigate the anti‐amnesic‐like effect of *K. crenata* extract on D‐galactose‐treated rats and possible mechanisms of action. Compared with donepezil, treatment with the aqueous extract significantly attenuated memory impairment (long‐ and short‐term memory), suggesting an anti‐amnesic effect. Analysis of possible mechanisms of action revealed that these effects are likely mediated by antioxidant and anti‐inflammatory properties, suggesting neuroprotective activity. This justifies its empirical to treat dementia and neurological diseases in Cameroonian folk medicine.

## Author Contributions

A.K.K., S.K., and C.M. designed the study, performed the data analysis, and wrote the manuscript. S.T.M., N.B., S.K., and A.K.K. critically reviewed the manuscript for important intellectual content. All authors read and approved the final manuscript.

## Ethics Statement

All procedures were performed according to the guidelines of the National Ethics Committee of Cameroon (Ref. No. FW‐IRB00001954, October 22, 1987).

## Conflicts of Interest

The authors declare no conflicts of interest.

### Peer Review

The peer review history for this article is available at https://publons.com/publon/10.1002/brb3.70261

## Data Availability

The datasets used and analyzed during the current study are available upon simple request from the corresponding author.
